# Impact of aeration change and beam arrangement on the robustness of proton plans

**DOI:** 10.1002/acm2.12503

**Published:** 2019-02-12

**Authors:** Nadya Shusharina, Barbara Fullerton, Judy A. Adams, Gregory C. Sharp, Annie W. Chan

**Affiliations:** ^1^ Department of Radiation Oncology Massachusetts General Hospital Harvard Medical School Boston MA USA; ^2^ Department of Otolaryngology Massachusetts Eye and Ear Infirmary Harvard Medical School Boston MA USA

**Keywords:** adaptive radiation therapy, nasopharyngeal carcinoma, paranasal sinus cancer, proton

## Abstract

This study determines the impact of change in aeration in sinonasal cavities on the robustness of passive‐scattering proton therapy plans in patients with sinonasal and nasopharyngeal malignancies. Fourteen patients, each with one planning CT and one CT acquired during radiotherapy were studied. Repeat and planning CTs were rigidly aligned and contours were transferred using deformable registration. The amount of air, tumor, and fluid within the cavity containing the tumor were measured on both CTs. The original plans were recalculated on the repeat CT. Dosimetric changes were measured for the targets and critical structures. Median decrease in gross tumor volume (GTV) was 19.8% and correlated with the time of rescan. The median change in air content was 7.1% and correlated with the tumor shrinkage. The median of the mean dose *D*_mean_ change was +0.4% for GTV and +0.3% for clinical target volume. Median change in the maximum dose *D*_max_ of the critical structures were as follows: optic chiasm +0.66%, left optic nerve +0.12%, right optic nerve +0.38%, brainstem +0.6%. The dose to the GTV decreased by more than 5% in 1 case, and the dose to critical structure(s) increased by more than 5% in three cases. These four patients had sinonasal cancers and were treated with anterior proton fields that directly transversed through the involved sinus cavities. The change in dose in the replanning was strongly correlated with the change in aeration (*P* = 0.02). We found that the change in aeration in the vicinity of the target and the arrangement of proton beams affected the robustness of proton plan.

## 1 INTRODUCTION

Accounting for changes in patient anatomy during the radiation treatment remains one of the main challenges in the medical physics of radiation therapy.^1^ The high conformity of the proton beam allows one to spare critical structures at all dose levels with acceptable homogeneity within the target volume.^2^ As a result, proton beam therapy for the nasal cavity and paranasal sinuses has been shown to have very promising outcome with potential lower toxicity than photon therapy.^3^, ^4^, ^5^, ^6^, ^7^ However, the same characteristics that make protons attractive may result in high sensitivity of the proton beam to anatomic changes occurring during treatment.

Malignancies of the nasal cavity, paranasal sinuses, and nasopharynx are located in areas of the skull base with variable amount of air and soft tissue densities. Variations in air and fluid content in the nasal cavity and paranasal sinuses during the course of radiotherapy could affect the proton dose distribution. This may lead to a change in dose to target coverage and/or normal structures. Late toxicities such as brain injury, cerebrospinal fluid leakage, and vision loss has been reported for patients with head and neck cancer patients treated with proton or carbon therapy.^5^, ^8^, ^9^

In photon treatments, dosimetric change during treatment is mainly caused by the change in position of the tumor and critical structures relative to planned treatment.^10^, ^11^ In contrast, the sharp fall‐off of proton makes protons very sensitive to variations in treatment depths within patients. Reduction in tumor and clearing or opacification of sinuses may result in shift of the high dose deposition, potentially lead to change in dose to the targets and critical structures. Moreover, proton therapy is more susceptible than photon therapy to tissue density heterogeneities as proton range is density‐dependent.

In this study, we investigated the impact of change in tumor size and aeration in the skull base as well as beam arrangement on the robustness of passive‐scattering proton therapy plans and determined the importance of adaptive proton planning.

## 2 MATERIALS AND METHODS

This study was performed with approval of the Institutional Review Board of Massachusetts General Hospital and in accord with an assurance filed with and approved by the U.S. Department of Health and Human Services.

### 2.A Study design

Between March 2009 and September 2012, 14 consecutive patients with locally advanced sinonasal malignancy or nasopharyngeal carcinoma who underwent a repeat planning CT during proton treatment were included in the study. Repeat scan was acquired between the 19th and 31st fraction of the treatment course. Dosimetric analysis was performed by comparing planned dose distribution with the distribution calculated on repeat CT. Patient and tumor characteristics are summarized in Table 1.

**Table 1:** Patient and tumor characteristics

Pat no.	Age	Gender	Histology	Location	TNM	GTV (cc)	Fract. no (dose, Gy) at rescan	Aeration change, %	GTV decrease, %
1	63	M	SCC	Nasal cavity	T4N2M0	12.18	19 (38)	4.7	11.7
2	31	F	NK/TCL	Nasal cavity	T4N0M0	72.3	21 (42)	3.1	6.8
3	25	M	NPC	Nasopharynx	T4N1M0	93.56	21 (42)	6.2	7.1
4	42	M	NPC	Nasopharynx	T2N3M0	16.32	24 (48)	14.7	28.1
5	59	M	SCC	Right maxillary sinus	T4N0M0	54.72	25 (50)	10.2	9.4
6	69	M	NPC	Nasopharynx	T4N2M0	89.87	26 (52)	5.0	14.6
7	57	F	NPC	Nasopharynx	T4N2M0	50.66	27 (54)	9.9	22.4
8	47	M	SCC	Nasal cavity	T1N0M0	7.35	27 (54)	2.1	18.9
9	56	M	ACC	Sphenoid sinus	T4N0M0	38.8	27 (54)	9.9	10.8
10	68	M	SCC	Nasal cavity	T4N0M0	62.74	28 (56)	2.2	21.6
11	67	M	NPC	Nasopharynx	T3N1M0	109.15	28 (56)	11.0	21.9
12	54	F	MPNST	Right maxillary sinus	T2N1M0	0.34	29 (58)	3.8	20.6
13	45	M	SCC	Ethmoid sinus	T4N0M0	31.09	30 (60)	8.0	43.9
14	49	M	SCC	Ethmoid sinus	T4N2M0	124.55	30 (60)	18.0	34.2
Mean	52.4					54.55	26 (52)	7.6	19.0
Std	13.4					39.49	3.5 (6.9)	4.6	10.2
Median	55.5					54.72	27 (54)	7.1	19.8
Min	25					0.34	19 (38)	1.7	8.8
Max	69					124.55	30 (60)	18.0	43.9

Aeration change was calculated by measuring air content in the cavity that contained the tumor and the involved sinuses on pretreatment and repeat CT scans.

SCC, squamous cell carcinoma; NK/TL , NK/T cell lymphoma; NPC, nasopharyngeal carcinoma; ACC, adenoid cystic carcinoma; MPNST, malignant peripheral sheath tumor; TNM, Tumor Nodes Metastasis; GTV, gross tumor volume; cc, cubic centimeters.

### 2.B Treatment

Patients were treated with proton radiotherapy and concurrent cisplatin‐based chemotherapy, except for one patient who received radiotherapy only (patient #12 in Table 1). The total dose to the gross tumor volume (GTV) was 70 Gy(RBE) over 35 fractions except for one patient with NK/T cell lymphoma who received 62 Gy over 31 fractions (patient #2 in Table 1). Passively scattered treatment plans were generated using treatment planning system (XiO, version 2.4, Elekta AB Stockholm, Sweden) that is clinically commissioned for proton radiotherapy at our institution.

During treatment, the patients were immobilized with a thermoplastic mask and a head cup to assure a proper and repeatable position of the head. Treatment plans utilized a set of beams for the clinical target volume (CTV) and another set for the GTV. The number and the direction of the beams employed depended on the location and the extent of the tumor as well as the physical relationship between the tumor and normal structures. In general, for patients with nasopharyngeal cancer, a pair of anterior obliques or lateral fields coupled with a posterior field was used when the tumor is confined to the nasopharynx. When the tumor in the nasopharynx extended superiorly to the skull base, superior anterior oblique fields were added to allow sparing of the optic structures. For patients with sinonasal cancer, combination of beams including superior anterior oblique(s), anterior oblique(s), posterior oblique(s), and/or laterals were used, depending on the location and extent of tumor involvement. In general, multiple beams that transverse through the sinus cavities that contained the tumor were employed.

In this study, robustness of the proton plans was achieved by adding the following margins: a 3.5% +1 mm was added to the range, a compensator smearing of 3 mm was applied to account for setup uncertainty, and an aperture margin of 8 mm to account for the lateral penumbra and setup uncertainty. For each individual beam, a brass aperture was used to achieve lateral shaping whereas a compensator was used for distal shaping. A patch combination (split‐target volume) technique was used to optimize the proton dose distribution within an irregular volume in close proximity to critical normal structures.^12^ The target volume was divided into multiple segments that were optimized and treated by a separate radiation fields, referred to as “patch field” and “through field”. Utilizing the sharp dose fall‐off of the Bragg peak, distal fall‐off 50% dose of the patch field was matched with the lateral penumbra's 50% dose of the through field. To ensure that the magnitude of the low and high dose along the path line is acceptable, a combination of patch fields with different junctions was used. Orthogonal kV setup films and kV field portals were acquired daily before treatment. Proton treatments were delivered at the MGH Francis H. Burr Proton Therapy Center using 230 MeV beams.

### 2.C Anatomical analysis

High‐resolution contrast‐enhanced CT images of 1.25–2.5 mm thickness were used for delineation of targets and normal structures. Contouring was performed using commercial software (MIM Maestro, version 6, MIM Software, Cleveland, OH). The contours of all normal structures were drawn or verified by the experienced neuro‐anatomist in the CT scans for each patient. The neuroanatomical structures in the skull base that were contoured included the retinas, optic nerves, optic chiasm, brainstem, frontal lobes, temporal lobes, cochleas, lenses, and lacrimal glands. In addition, the nasal cavity and the paranasal sinuses (frontal, ethmoid, maxillary and sphenoid) were also delineated. A composite cavity containing the tumor and the involved sinuses was defined and contoured. The amount of air, tumor, and fluid within the composite cavity was measured on pretreatment and repeat CT using analysis tool provided in the commercial software (MIM Maestro, version 6, MIM Software, Cleveland, OH).

### 2.D Image registration

All planning CT scans were first registered rigidly with the replanning CT to match the spatial positions. The alignment was done with respect to bony structures of the skull. Deformable registration was then performed between the rigidly transformed repeat CT and the planning CT to transfer the delineated structures onto the replanning CT.

### 2.E Statistical analysis

Tumor shrinkage was evaluated for association with increase in aeration in the skull cavities containing tumor. The doses to the tumor target and OARs calculated on the planning and repeat CT scans were compared to find statistically significant difference. The data were analyzed by means of Pearson's correlation coefficient. One‐tailed *t*‐test with *P* < 0.05 indicated statistical significance.

## 3 RESULTS

### 3.A Tumor shrinkage

**Figure 1:**
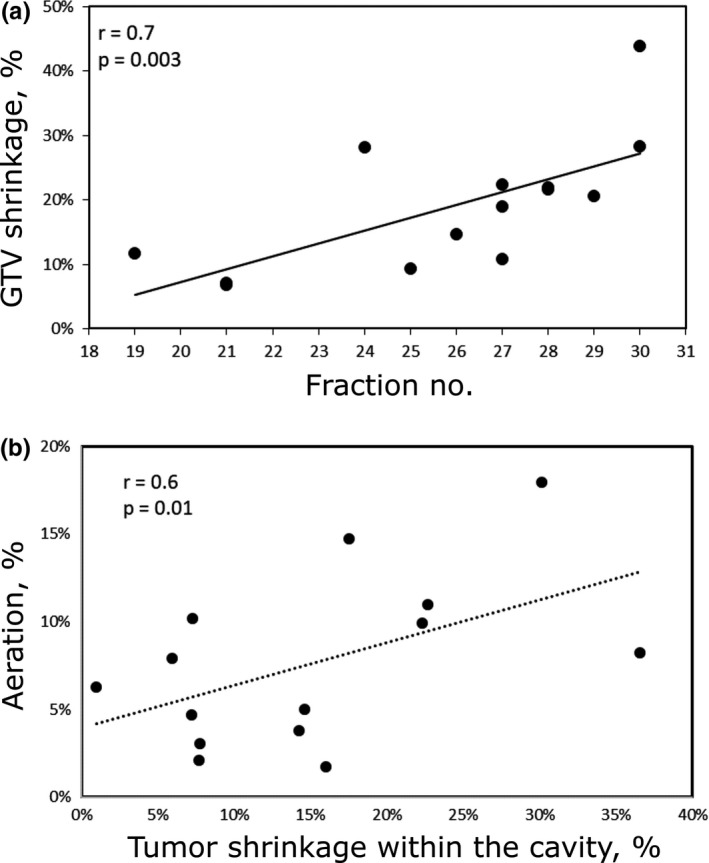
(a) Percentage of gross tumor volume (GTV) shrinkage at the fraction of rescan for each patient; (b) aeration change in the cavity containing the tumor as a function of percent of tumor shrinkage.

The median decrease in the GTV of the primary site was 19.8% (range: 6.8–43.9%). We found a statistically significant correlation between the time of rescan and the extent of tumor shrinkage (*r* = 0.7, *P* = 3 × 10^−3^) [see Fig. 1(a)]. For patients who were rescanned at or before 5 weeks, the median decrease in the primary GTV was 9.4% (*P* = 0.25) compared to 28.3% (*P* = 0.006) for patients who were rescanned after 5 weeks.

### 3.B Change in aeration

**Figure 2:**
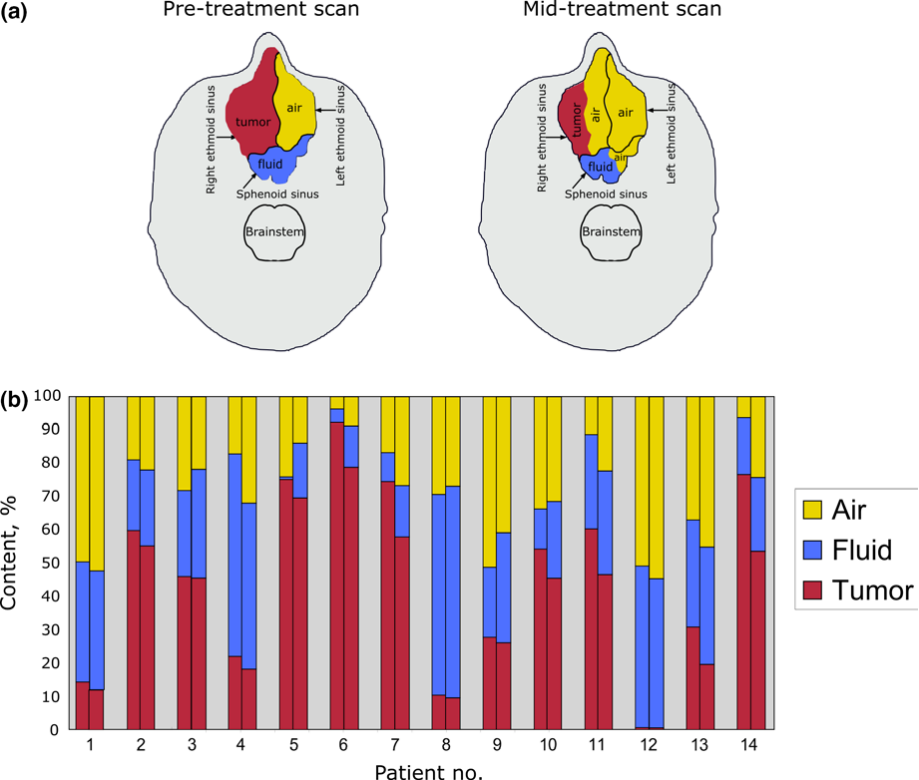
(a) Schematic representation of a relative change in tumor, fluid, and air volumes in the paranasal sinuses encompassing the tumor. The shrinkage of tumor in the right ethmoid sinus has resulted in increased aeration in the right ethmoid sinus. Due to improved drainage as a result of tumor shrinkage, there was also increased aeration of the sphenoid sinus posteriorly. (b) Tumor, fluid, and air content measured on pretreatment (first column) and repeat (second column) CTs for each patient.

In Fig. 2(a), we show schematically the change in relative sizes of the tumor, fluid, and air content within the composite cavity containing the tumor over the course of treatment. The chart below Fig. 2(b) shows that tumor in the cavity can be replaced by air, fluid, or combination of air and fluid.

We are particularly interested in the extent of increased air content in the nasal and paranasal cavities during treatment. Any Increase in aeration in the composite cavity during treatment could result in over‐shooting of proton beam and therefore over‐dosing of the surrounding critical structures. We have shown that the change in aeration in the composite cavity correlated significantly with the extent of tumor shrinkage (*r* = 0.6, *P* = 0.01) [see Fig. 1(b)]. However, the air/fluid ratio could change independently of tumor shrinkage. The median change in air content of all patients was 7.1% (range: 1.7–18%). In six cases, the tumor was replaced by both air and fluid. In three cases, an increase in aeration within the composite cavity was observed due to tumor shrinkage and fluid clearing. For these three cases, the increase in air volume was 4.7, 14.8 and 3.8%, respectively.

### 3.C Other anatomical changes

We also observed that the size of the surgical flap, the tissue used to repair surgical defects, changes during the course of radiation. For the one patient with a myocutaneous free flap in the nasal cavity, a decrease of 4.7% in the volume of the flap was observed during treatment [see Fig. 4(c)]. In another patient with a primary tumor in the nasal cavity, an increase in size of the soft tissue overlying the anterior nasal cavity (0.25 cm anterior‐posterior and 0.5 cm laterally) was observed (Fig. S2b). In both of these cases, dosimetric changes due to these anatomical cases were observed.

### 3.D Dosimetric changes

#### 3.D.1 Impact of aeration on target coverage

**Figure 3:**
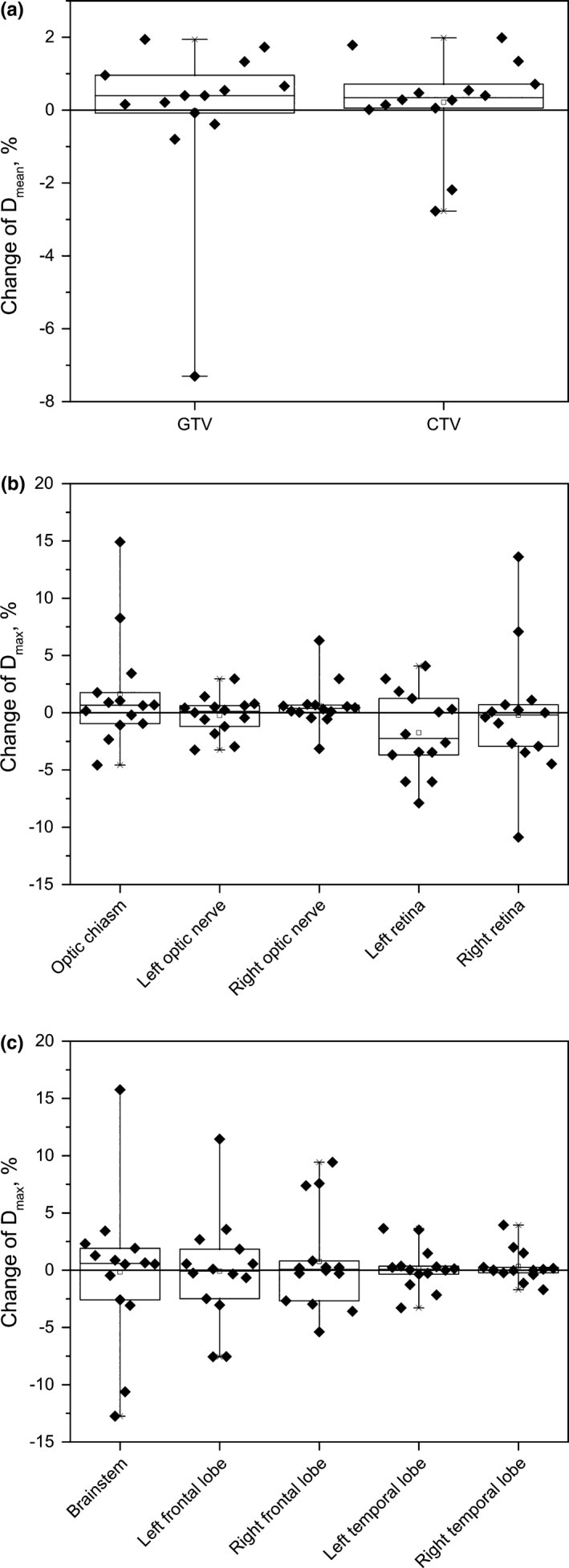
Box plots of percentage difference of the doses calculated on planning and repeat CT; (a) for gross tumor volume (GTV) and CTV; (b) for optic structures; (c) for the brainstem and brain lobes. Shown in each plot are 25th and 75th percentile (box edges), the median (solid line within box) and most extreme values (whisker edges). The solid line on each panel indicates no change in dose.

**Figure 4:**
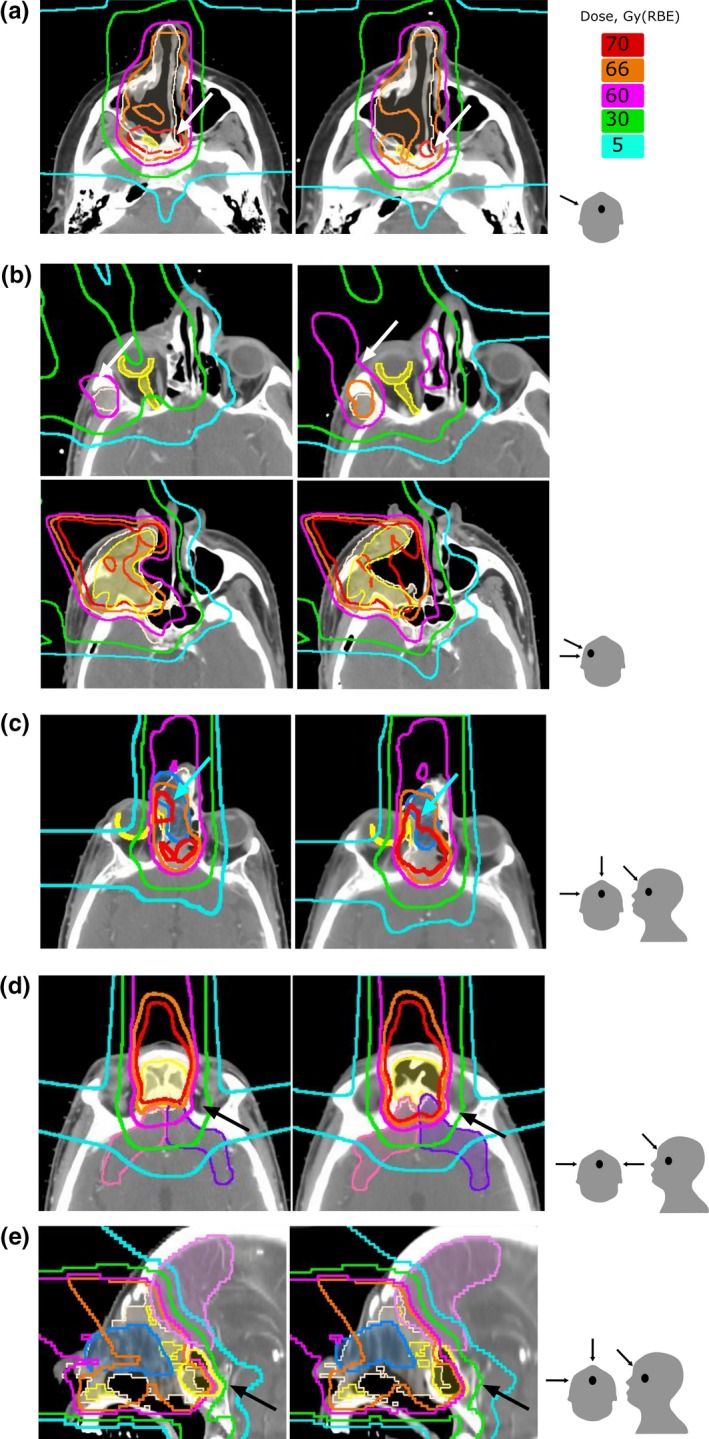
Comparison of dose distributions calculated on planning CT (left) and repeat CT (right). gross tumor volume (GTV) and CTV are shown in shaded yellow and white color, respectively. Panel (a) shows aeration increase in the right maxillary sinus and a shift of the dose away from GTV (patient #12). Panel (b) (upper row) shows an increased dose to the right retina and optic nerve. Panel (b) (lower row) shows decrease in GTV and CTV coverage due to increase in soft tissue in the posterior wall of the right maxillary sinus (patient #5). Panel (c) shows increase in the dose to the right retina due to shrinkage of the surgical flap (patient #1). Panel (d) shows increase in the dose to the frontal lobes due to clearing of the bilateral frontal sinus (patient #14). Panel (e) shows increase in the dose to a frontal lobe due to fluid clearing in the sphenoid sinus and shrinkage of the surgical flap (patient #1). The last column demonstrates the direction of the boost fields.

To quantitate the difference in dose distributions calculated on planning and repeat CTs, we measured the corresponding change in the mean dose *D*_mean_ for the GTVs and CTVs and the maximum dose *D*_max_ for optic and brain structures relative to the prescribed doses (Fig. 3). The absolute doses for the targets and critical structures are given in Fig. S1. There was no statistically significant decrease in the dose to either GTV or CTV across the entire patient set (*P* = 0.99 and 0.86 for the GTV and CTV, respectively). The median change to the mean dose was 0.4% (range: −7.3 to 1.9%) to GTV and 0.3% (range: −2.8 to 1.8%) to CTV. In one outlier (patient #12 in Table 1 with sinonasal cancer), the mean dose of the GTV decreased by 7.3% due to clearing of the maxillary sinus. Figure 4(a) shows a shift of 70‐Gy(RBE) isodose line away from GTV in this patient. As a result, the GTV was partially covered by 66 Gy instead of 70 Gy(RBE) and the CTV coverage did not change.

To quantitate the target coverage, we calculated the volume that received 95% of the prescribed dose (V_95_) for CTV and GTV. In the original plans, the median V_95_ for GTV on the original plan was 98.5% (range: 87.8–100%) and the V_95_ for CTV was 99.9% (range: 98.6–100%). On the recalculated plans, the median V_95_ for GTV was 98.2% (range: 18.1–100%) and the median V_95_ for CTV was 99.8% (range: 97.8–100%). The median change between the original and recalculated plans was −0.04% (range: −1.56 to 0.05%) and −0.06 (range: −81.9 to 2.86%) for the GTV and CTV, respectively.

#### 3.D.2 Impact of aeration on the doses to normal structures

Changes in the maximum dose to the optic structures (optic chiasm, optic nerves and retinas) were not statistically significant. The median changes and the range of changes for these structures were 0.66% (−4.6 to 14.9%) for the chiasm (*P* = 0.88), 0.12% (−3.2 to 3%) (*P* = 0.98) and 0.38% (−3.1 to 3%) (*P* = 0.95) for the left and right optic nerves, respectively; and −2.2% (−7.9 to 4%) (*P* = 0.89), and −0.2% (−10.9 to 13.6%) (*P* = 0.98) for the left and right retinas, respectively.

There were two patients for whom the changes might be considered clinically significant. For one patient (patient #5 in the Table 1 with sinonasal cancer), the *D*_max_ increased by 14.9% for the optic chiasm, 6.3% for the right optic nerve, and 13.6% for the retina. For this patient, the decrease in aeration of the right maxillary sinus resulted in over‐shoot of the proton beam. Although the initial plan was created to spare the optic structures on the right side, the sparing was compromised due to the density variation around surgically removed maxillary sinus. Dose distributions for this case are shown in Fig. 4(b). The upper panel shows an increase in dose around orbital/optic structures and the lower panel shows the change in the dose distribution due to increase in the soft tissue density in the posterolateral wall of the right maxillary sinus. For the other patient (patient #1 in the Table 1 with sinonasal cancer) for whom the anatomical change during course of radiation might result in significant clinical impact, the changes *D*_max_ were +8.3% for the optic chiasm and +7.1% for the right retina due to 4.7% decrease in size of the forehead flap [Fig. 4(c)].

The maximum dose to the frontal and temporal lobes did not change significantly for most patients. Median changes and ranges were: −0.1% (range: −7.6 to 11.4%) for the left frontal lobe, 0.1% (−5.4 to 9.4%) for the right frontal lobe, 0.1% (−3.3 to 3.6%) for the left temporal lobe, 0.01% (−1.7 to 3.9%) for the right temporal lobe. There was a case in which *D*_max_ increased by 11.4% for the left frontal lone and by 9.4% for the right frontal lobe (patient #14 in the Table 1). The increase in frontal lobes was associated with complete clearing of the opacification of fluid in the frontal sinuses (see [Fig. 4(d)].

In two cases, *D*_max_ for the right frontal lobe increased by 7.6% (patient #1 in the Table 1) and 7.4% (patient #5 in the Table 1 with sinonasal cancer), respectively. The increase by 7.6% was caused by a combination of shrinkage of the surgical flap and clearing of opacification in the sphenoid sinus [Fig. 4(e)], and the 7.4% increase was caused by the density change around surgically removed maxillary sinus.

The median maximum dose change to the brainstem was 0.6% (range: −12.7 to 15.8%). For one patient, for whom the observed increase in the maximum dose was 15.8%, the planned maximum dose was 18.3 Gy(RBE), the lowest among all patients and therefore this increase was not considered as clinical significant.

**Figure 5:**
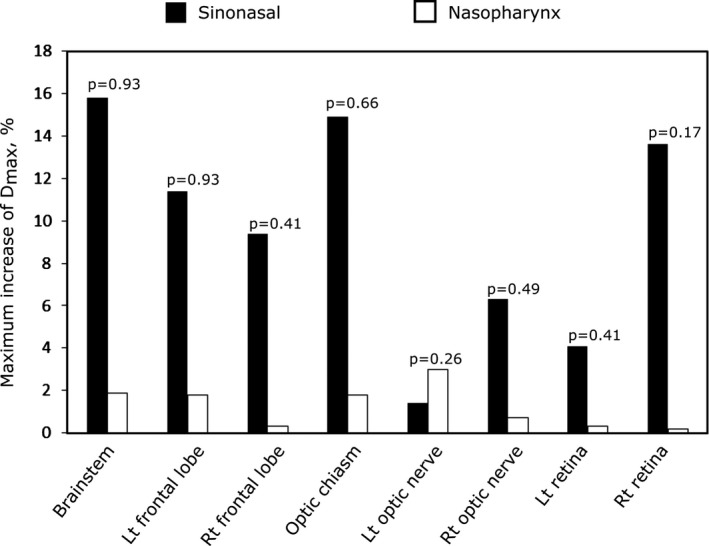
Maximum change in *D*_max_ to the brain structures and optic structures for the sinonasal cancer (black columns) and nasopharyngeal carcinoma (white columns).

**Figure 6:**
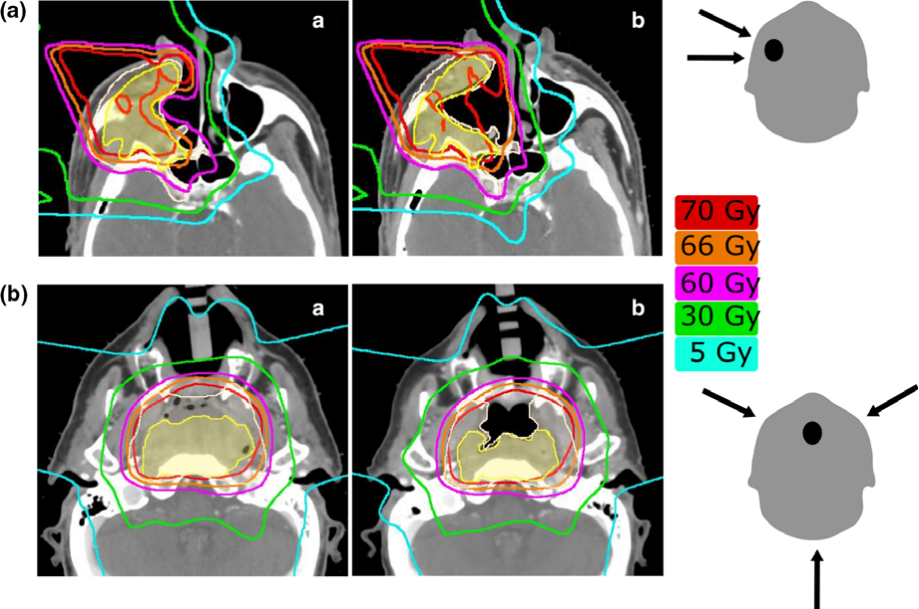
Radiation dose distributions for (a) patient with right‐sided sinonasal tumor treated with a right lateral and a right‐anterior oblique field for the boost; (b) Patient with nasopharyngeal centered tumor treated with a right‐anterior oblique, a left‐anterior oblique, and a posterior beam. (a) and (b) show pretreatment and repeat scans, respectively. Aeration change was approximately the same for the two patients; in (a) it was decreased by 10% and in (b) it increased by 11%. GTV and CTV are shown in shaded yellow and white color, respectively. The last column shows the direction of the boost fields.

We identified four patients (#1, 5, 12, and 14 in the Table 1) for whom the dose distributions were compromised justifying a need for adaptive replanning. All these patients were treated for a sinonasal cancer with the primary tumor in nasal cavity (patient #1), maxillary sinus (patient # 5 and #12), and ethmoid sinus (patient #14). The change in aeration for these patients was 4.7, 10.2, 3.8, and 18.0%, respectively. All of these patients were treated with a combination of anterior, anterior oblique, ±lateral fields for the GTV boost that transversed through the nasal and sinus cavities. Figure 5 compares maximum change in *D*_max_ of the optic/orbital and brain structures for the patients with sinonasal and nasopharyngeal malignancies. The change in the planned dose to the critical structures was higher for sinonasal cancer patients than for nasopharyngeal cancer patients (see Fig. 5). We have found that the extent of dosimetric change as a result of change in aeration depends on the direction and the number of proton beams. In general, treatment plans that involve more proton beams and beams that do not transverse on the sinuses such as in the case of nasopharyngeal cancer (Fig. 6) exhibit less dosimetric change compared to those that involve fewer beams and with beams that transverse on the sinuses such as in the treatment of sinonasal cancer [Fig. 6(a)].

## 4 DISCUSSION

In this study, we investigated the impact of anatomical changes during proton radiation and beam arrangement on the robustness of proton plans. We focused on dosimetric changes that might justify a need for adaptive proton planning. As it is well known, the proton ranges are very susceptible to changes in air cavities within radiation fields.^13^, ^14^ Nevertheless, our study has shown that in most of the cases the coverage of target volumes was not compromised substantially even for the patients that were rescanned late in the course of treatment and for those who exhibited a pronounced change in aeration within the cavity. This indicates that the treatment planning technique that utilizes multiple beams and field patching technique often results in robust proton beam plans.

The major dosimetric consequences that affect critical structures occur when the amount of air within the irradiated cavity increases or decreases and the high dose deposition shifts forward or backward in the direction of the beam. In three cases, the dose to brainstem deviated from planned by more than 5%. For these patients, all were treated with proton beams that transversed through the sinus cavities—the most common for treatment of sinonasal cancers. The plans that did not involve beams that transverse through the sinus cavities were the most robust to change in aeration. In the case of posterior beams, a change in aeration affected only the exit dose and therefore the dose distribution was not substantially compromised. This may explain the fact that we observed alteration in dose distribution due to aeration change mainly in sinonasal cancer and not in nasopharyngeal carcinoma (Fig. 6). In the treatment of sinonasal cancer, a set of anterior or anterior oblique fields is predominantly used whereas in the treatment of nasopharyngeal carcinoma, posterior fields in addition to the anterior oblique fields are always employed.

Similar findings were recently published for the cohort of 20 patients treated with proton therapy for nasal cavity or paranasal sinus cavities.^14^ The air content in the cavities increased in 18 out 20 cases. An increase in the dose to the brainstem beyond 60 Gy was observed for 3 patients, and for 10 patients the dose to the optic chiasm increased beyond 50 Gy. Our study, however, shows that proton beam arrangement is as important, if not more important, than change in aeration on the robustness of proton plan.

## 5 CONCLUSIONS

We found that there was no statistically significant decrease in the dose to either GTV or CTV across the entire patient set. For four out of fourteen patients the dose to the critical structures was compromised due to change in aeration near the target justifying a need for adaptive replanning. We also found that shrinkage of the surgical flap can compromise the planning dose distribution. In summary, adaptation during proton treatment may be needed for selected patients whose plans rely heavily on beams that transverse through the sinus cavities.

In our current clinical practice, patients with sinonasal and nasopharyngeal malignancies are routinely rescanned at least once during their course of proton treatment. Patients with change in aeration and tumor volume will be treated with the new proton plans. Prospective studies with a larger patient set and weekly CT rescans for each patient are necessary to determine the criteria for appropriate cause and time of replanning.

## CONFLICT OF INTEREST

The authors declare no conflict of interest.

## Supporting information


**Fig. S1.** Results from two plans, based on planning CT (black columns) and on repeat CT (white columns).
**Fig. S2.** Comparison of dose distributions calculated on planning CT (left) and repeat CT (right).
