# Supplementary material for: Impact of aeration change and beam arrangement on the robustness of proton plans
Source: J Appl Clin Med Phys. 2019 Feb 12;20(3):14–21. doi: 10.1002/acm2.12503 (PMC6414139; doi:10.1002/acm2.12503)
Supplement: Supplementary file 1 — Fig. S1. Results from two plans, based on planning CT (black columns) and on repeat CT (white columns). Fig. S2. Comparison of dose distributions calculated on planning CT (left) and repeat CT (right). [file ACM2-20-14-s001.pdf]

Supplementary materials

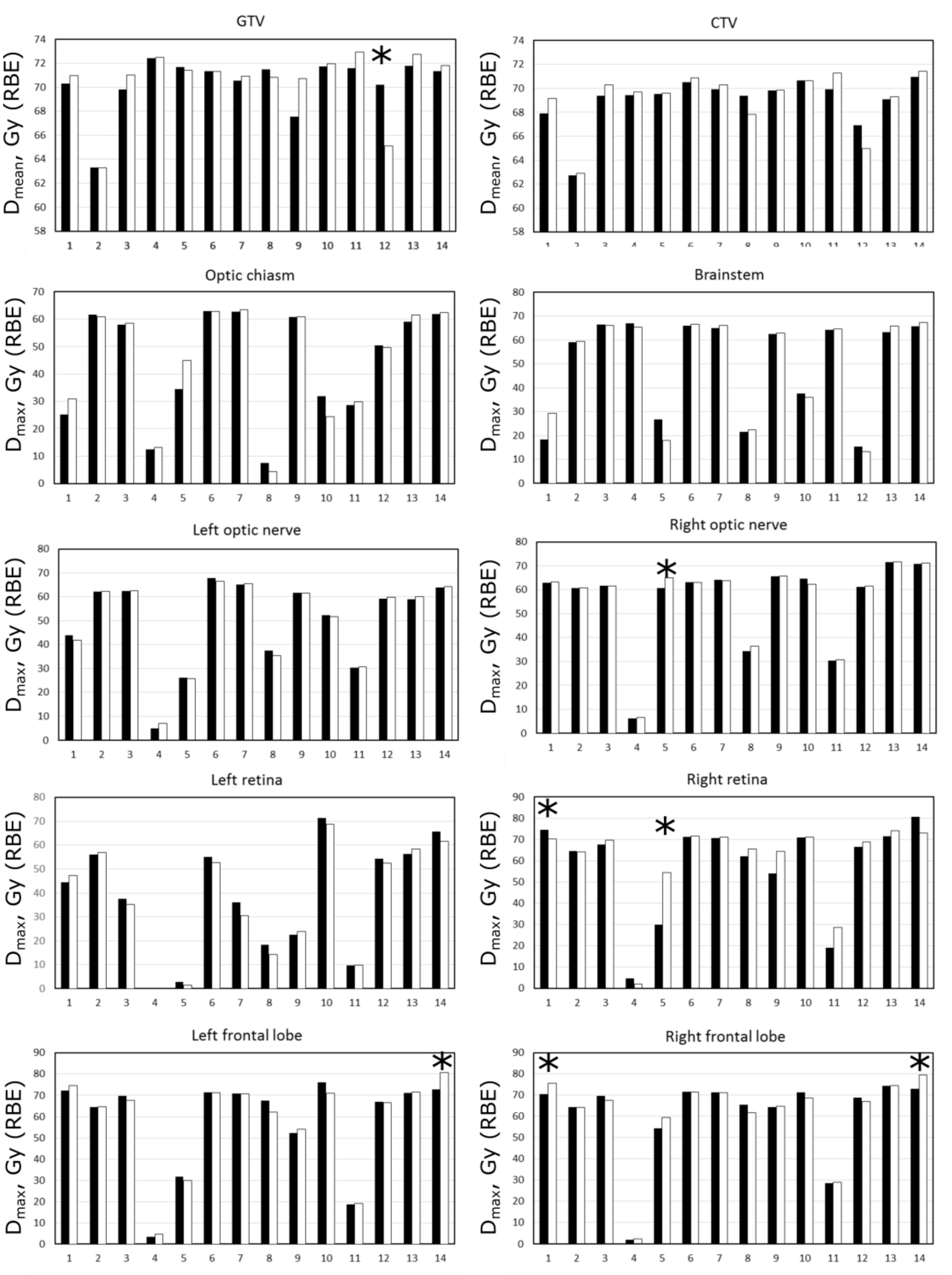

Figure S1. Results from two plans, based on planning CT (black columns) and on repeat CT (white columns). D_{mean} for the targets and D_{max} for the critical structures are shown for all patients. The patients with clinically significant change of the dose are marked with an asterisk.

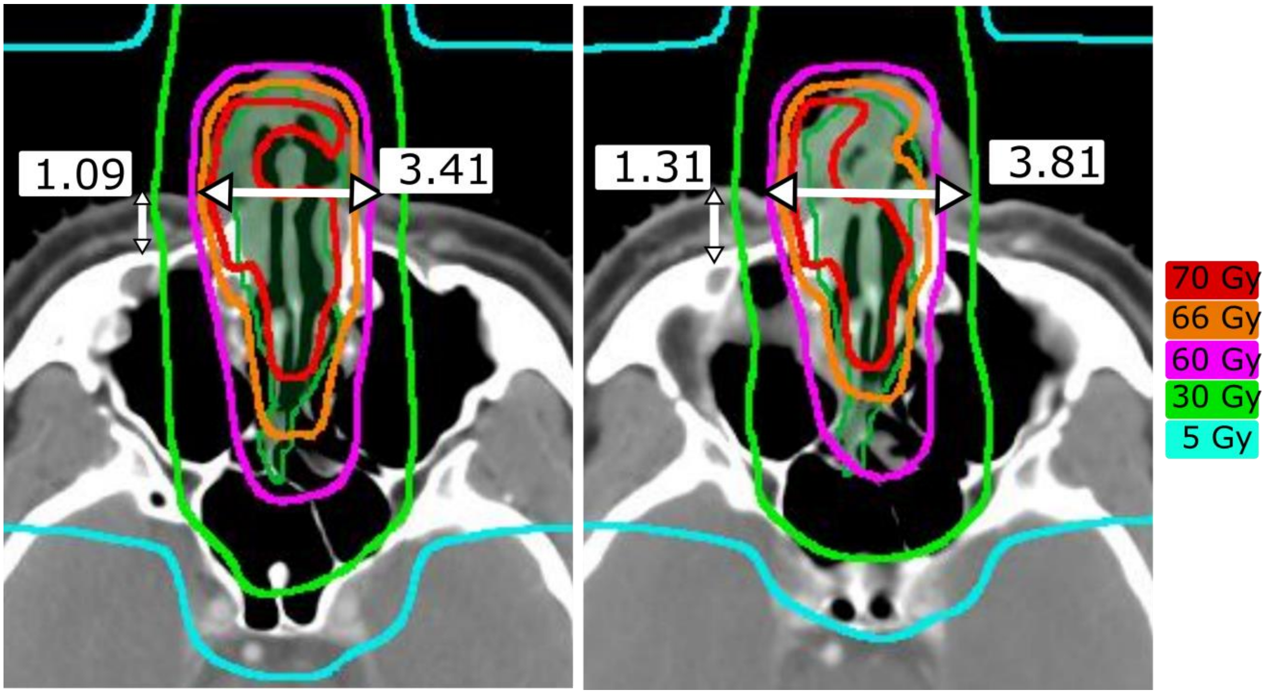

Figure S2. Comparison of dose distributions calculated on planning CT (left) and repeat CT (right). Facial swelling at the time of rescan shown by measuring lines (with the measures in cm) causes a redistribution of the high dose in the upper part of the CTV shown by a green filled area.
